# Behavior in a stressful situation, personality factors, and disease severity in patients with acute myocardial infarction: baseline findings from the prospective cohort study SECAMI (The Secondary Prevention and Compliance following Acute Myocardial Infarction-study)

**DOI:** 10.1186/1471-2261-11-45

**Published:** 2011-07-21

**Authors:** Lena André-Petersson, Mona Schlyter, Gunnar Engström, Patrik Tydén, Bo Hedblad

**Affiliations:** 1 Department of Clinical Sciences, Lund University, Skåne University Hospital, Malmö, Sweden; 2 Department of Neurology, Skåne University Hospital, Malmö, Sweden; 3 Department of Cardiology, Lund University, Skåne University Hospital, Malmö, Sweden

## Abstract

**Background:**

Psychosocial stress has been identified as a risk factor in association with cardiovascular disease but less attention has been paid to heterogeneity in vulnerability to stress. The serial Color Word Test (CWT) measures adaptation to a stressful situation and it can be used to identify individuals that are vulnerable to stress. Prospective studies have shown that individuals with a maladaptive behavior in this test are exposed to an increased risk of future cardiovascular events. The aim of the present study was to investigate whether maladaptive behavior in the serial CWT alone or in combination with any specific personality dimension was associated with severity of myocardial infarction (MI).

**Methods:**

MI-patients (n = 147) completed the test and filled in a personality questionnaire in close proximity to the acute event. The results were analyzed in association with four indicators of severity: maximum levels above median of the cardiac biomarkers troponin I and creatine kinase-MB (CKMB), Q-wave infarctions, and a left ventricular ejection fraction (LVEF) ≤ 50%.

**Results:**

Maladaptive behavior in the serial CWT together with low scores on extraversion were associated with maximum levels above median of cardiac troponin I (OR 2.97, CI 1.08-8.20, p = 0.04) and CKMB (OR 3.33, CI 1.12-9.93, p = 0.03). No associations were found between the combination maladaptive behavior and low scores on extraversion and Q-wave infarctions or a decreased LVEF.

**Conclusions:**

Maladaptive behavior in combination with low scores on extraversion is associated with higher cardiac biomarker levels following an MI. The serial CWT and personality questionnaires could be used to identify individuals vulnerable to the hazardous effects of stress and thereby are exposed to an increased risk of a more severe infarction.

## Background

During recent decades, rates of mortality from coronary heart disease (CHD) have steadily declined in Sweden as in many other Western European countries and Northern America [1,2]. A suggested explanation for the decline is that severity of acute myocardial infarction (AMI) has been reduced as a consequence of not only treatment procedures but also by primary prevention efforts [3]. Psychosocial stress in relation to AMI [4] has made it necessary to include the concept of stress as a risk factor that should be targeted in primary prevention programs.

Stress is subjective because it reflects a dynamic relationship between an individual and the environment. Coping strategies are used to modify psychological and physiological reactions of stress and are generally thought to have a buffering effect on stress. The coping process includes behavioral efforts but also cognitive dimensions because action is preceded by an appraisal of both the situation at hand and of available coping resources [5]. Individuals react differently in response to different stimuli depending on situation, nature of the stressor, and predisposition. Different coping responses have different physiological correlates. Active coping has been related to increased catecholamine release and sympathetic activation whereas passive behavior has been related to corticosteroid secretion [6]. If a stress reaction takes place it is assumed that the coping strategy was not sufficiently efficient or that it was maladaptive. Maladaptive coping is a response that elicits or maintains a harmful physiological process and if this occurs repeatedly the probability of mortality and morbidity is assumed to increase [7]. Individual differences in ability to cope with stress could serve as an explanation as to why certain individuals are more vulnerable than others.

We have previously shown that it is possible to assess how an individual cognitively manages and adapts to a stressful situation by the use of the serial Color Word Test (CWT) [8]. The test was included in the cohort study "Men born in 1914" in Malmö [9] which was the first time the test was used in an epidemiological context. Our analyses showed that the semi-experimental application of the test could be a significant contributor when analyzing prospective cardiovascular risk. Hypertensive men who many years in advance of a clinical event had difficulties to adapt to the stressful test were exposed to an increased risk of a myocardial infarction (MI) [10] or stroke [11]. Ventricular arrhythmia [12] and asymptomatic atherosclerosis [13] were less important predictors among the men who managed to handle the stressful task in an adaptive manner. Postinfarction mortality within 28 days and within one year could also be explained by behavior in the serial CWT [14]. Men with difficulties in the test were also less likely to benefit from a supportive network and were consequently exposed to an increased risk of a future MI [15].

Personality dimensions have been investigated in the pursuit of links between psychology and vulnerability in association with CHD. Personality can be defined as "those characteristics of the person that account for consistent patterns of feeling, thinking, and behaving" (16, p. 4) and could thus be viewed as a factor explaining why certain behaviors are expressed when an individual interacts with the environment. The Type D personality [17] and hostility [18] are examples of personality traits that have been associated with cardiovascular morbidity and mortality. The tendency to show exaggerated cardiovascular responses in association with mental stress has been labeled cardiovascular reactivity and such increased reactivity may act as a risk factor for coronary artery disease (CAD) development [19]. Cardiovascular reactivity has been related to exaggerated blood pressure responses [20], progression of carotid artery atherosclerosis [21], and increased serum lipid concentrations [22]. The phenomenon is not observed in all individuals and it has therefore been proposed that cardiovascular reactivity bears associations with dispositional factors [23]. Differences in cardiovascular reactivity as a response to stress could thus also explain why certain individuals are more vulnerable than others.

Our knowledge of which individuals are exposed to the greatest risks needs to be improved if primary prevention of MI shall be efficient. Popular opinion associates stress with MI in general whereas our assumption is that certain individuals are more vulnerable than others when exposed to stress. Maladaptive behavior as reflected in the serial CWT has previously been identified as a risk for cardiovascular events and one objective of the current study will be to explore whether maladaptive behavior also is associated with severity of MI. The five factor model of personality distinguishes five factors of personality and gives measures on Neuroticism, Extraversion, Openness, Agreeableness, and Conscientiousness [24,25]. Because the personality trait Neuroticism is related to emotional instability, anxiety, and depressiveness this would be the trait most likely to be associated with severity of disease. A second objective will be to consider whether maladaptive behavior in combination with any of these personality dimensions is associated with severity.

## Methods

The Secondary Prevention and Compliance following Acute Myocardial Infarction (SECAMI)-study was designed as a patient-based prospective cohort study with an overall aim of identifying the psychosocial factors that increase the risk of mortality and determine compliance with treatment. The serial CWT was included at baseline and at the same time measures on personality were collected.

The chosen indicators of disease severity were the biochemical markers maximum troponin I and creatine kinase-MB maximum levels above medians [3,26]. In addition, we chose Q-wave infarction [26], and a left ventricular ejection fraction (LVEF) ≤ 50% [17,27] to indicate severity.

### Patients

The collection of baseline-data in the SECAMI-study started in 2002 and ended in 2005. At the Cardiology Department, Skåne University Hospital, Malmö, approximately 630 cases of acute MI are treated each year and about 240 of these patients are below the age of 71. All patients with a diagnosis of AMI (International Classification of Diseases (ICD) ICD-10 code I21) who had not reached their 71st birthday were invited to take part. The cut-off at 71 years was decided on because we did not want to expose elderly patients to a large collection of examinations in such close proximity to the acute event. Patients below the age of 71 who did nor want to participate or had communication problems were excluded. At times of research staff's absence AMI-patients could not be asked to participate which constitutes another exclusion factor. Data from medical charts and socioeconomic circumstances together with cultural background were recorded. Before discharge, each patient met a clinical psychologist who administered the serial CWT and the questionnaire on personality.

Four hundred patients were included in the SECAMI and 180 patients were able to participate in the serial CWT. Thirty-three patients were excluded because of non-participation in all or some of the examinations of interest to this study. The analyses, thus, include 147 patients with a mean age of 58.1 years (range 30-70 years) and 39 (26.5%) were women.

Written consent was obtained from each patient and The Ethical Committee of the Faculty of Medicine, Lund University, Sweden, had approved the study.

### Background characteristics

Background characteristics included educational level (less than nine years/nine years or more), whether the individual had immigrated (yes/no), was living alone (yes/no), and whether the individual was professionally active or employed (yes/no).

### Clinical characteristics

Patients were asked about smoking habits and current smoking was coded as such when smoking was acknowledged (independent of quantity). The artery interventions Percutaneous Coronary Intervention (PCI) and Coronary Artery Bypass Graft (CABG) that the patients had undergone during previous hospitalizations were asked for and were here treated as a single variable called "previous revascularization". Registration was also made of previous MI, treatment of hypertension, hyperlipidemia, angina pectoris, diabetes mellitus and level of body mass index (BMI). The Beck Depression Inventory (BDI) [28] was used to measure prevalent depression. A cut-off at 17 points or more was used to indicate moderate to severe levels of depression [29].

### Psychological factors

#### Behavior in the serial Color Word Test

The CWT is a semi-experimental way to measure how individuals adapt in a stressful situation [8]. It is a version of the Stroop test [30], originally designed for the study of interference, and it has in detail, complete with graphic illustrations, been described elsewhere [8,10,31,32]. In short, the test consists of color words printed in an incongruent color where the object is to name the color of the print and disregard the written word. Scoring is done by measuring the time it takes to complete the test. The cognitive adaptation is studied as a process through a series of five identical subtests [33]. The unfolding time-pattern is assumed to reflect how the individual generally adapts when confronted with a challenging and conflicting task [34].

The analysis of test scores is done by considering the temporal aspects in a two-dimensional analysis where linear change of time consumption, from beginning until end, constitutes the Regression-dimension. The Variability-dimension is created by the observations that show a simultaneous variation along the long-term progression but which are not captured by a straight line. Difficulties in adaptation, i.e. failures in finding successful strategies to master the conflict between naming the color and disregarding the printed word, are shown as time consumptions that increase and vary from beginning until end. These difficulties are exhibited as time-patterns with high variability in both the Regression- and the Variability-dimension [8] and this behavior is labeled *maladaptive*. Behaviors categorized as *adaptive *show stable or increasing time consumption throughout the test but do not show recurrent declines and increases in time consumption. The Variability-dimension was chosen for the present study because its associations with increased incidence of MI in men [10,12,13]. All categorization of behavior in this study is based on comparisons of test scores with reference-medians obtained from the 180 patients that were able to participate in the CWT in the SECAMI-study.

### Personality

The abbreviated version of the NEO Personality Inventory was used to measure personality traits [24,25]. This inventory gives measures on five basic dimensions of personality; Neuroticism, Extraversion, Openness, Agreeableness, and Conscientiousness. The results from each dimension were treated as dichotomous variables. In order to construct clear variables of personality we decided on cut-off points at the 67^th ^percentile or the 33^rd ^percentile instead of dichotomizing at the median value. A high level of neuroticism was scored at levels above the 67^th ^percentile. Low levels of extraversion, openness, agreeableness, and conscientiousness were scored at levels below the 33^rd ^percentile.

### Severity of MI

All four indicators of severity of MI were chosen to be dichotomized in order to make the results clear.

#### Troponin I

Troponin I is a sensitive and specific biochemical marker of myocardial damage measurable within 3-4 hours from the onset of an MI [26]. The peak value during the first 24 hours was obtained from the patient's medical record. Measures were obtained with an interval of six hours. Range 0.07-271.00; SD = ± 36.0; Md = 5,87. Levels above median were used to indicate severity [3].

#### Creatine kinase

Creatine kinase and its MB isoenzyme CKMB, is also a sensitive and specific biochemical biomarker of myocardial damage, measurable within 4-6 hours after onset of an MI [26]. The peak value during the first 24 hours was obtained from the patient's medical record. Measures were obtained with an interval of six hours. Range 1.00-457.00; SD = ± 92.9; Md = 44.0. Levels above median were used to indicate severity [3].

#### Pathological Q-wave

A pathological Q wave on the 12 lead ECG signifies abnormal electrical activity seen in several conditions of the heart [26]. The diagnosis was given if a new pathological Q-wave was observed on ECG-recordings during the hospital stay.

#### Left ventricular ejection fraction

LVEF is a key measure of the left ventricular systolic function of the heart. It is most commonly determined by a two-dimensional echocardiography. A decreased LVEF can be caused by an infarction and might lead to heart failure with clinical signs and symptoms like edema and dyspnea [27]. Patients with an LVEF ≤ 50% were considered to have a decreased function [17].

### Statistical analyses

Unadjusted odds ratios were calculated by means of logistic regression analyses for the background and clinical variables in association with the severity indicators maximum troponin I level above median, maximum CKMB level above median, Q-wave infarction, and a left ventricular ejection fraction LVEF ≤ 50%. Differences in mean levels of maximum troponin I and CKMB were compared between patients with adaptive and maladaptive behavior and between patients with different levels in the five personality dimensions by using the one-way ANOVA test. The Pearson chi-square test was used to analyze differences in distribution of adaptive and maladaptive behavior in the CWT and low versus high levels in the five personality dimensions between patients with and without Q-wave infarctions and between patients with and without an LVEF ≤ 50%. Personality dimensions with p ≤ 0.10 were decided to be eligible for the final analyses.

The multivariate analyses of whether behavior in the CWT together with a specific personality dimension was associated with severity of MI were performed by means of logistic regression. In these analyses the odds ratios for a severe infarction were calculated for four strata of patients defined in terms of adaptive/maladaptive behavior in the CWT respectively low/high level in the chosen personality dimension. The following parameters were eligible for inclusion in the multivariate analyses: age, sex, having immigrated to Sweden, educational level less than 9 years, history of MI, previous revascularization, treatment for hypertension, treatment for hyperlipidemia, treatment for angina pectoris, treatment for diabetes mellitus and level of BMI, living alone, current smoking, presently not employed or professionally active, and moderate to severe level of depression. Four different analyses were performed because we had four different indicators of severity and in each analysis only variables associated with the severity-indicator with p < 0.20 were included.

## Results

Background data and clinical characteristics of the included 147 patients are presented in Table 1 (Table 1). Eighteen (12.2%) patients had a previous experience of an MI, less than half (44.2%) had been prescribed treatment for hypertension, and approximately one fifth had been prescribed drugs to lower lipid levels (21.1%). Fifteen patients were considered to have a moderate to severe level of depression (10.2%).

**Table 1:** Background and clinical characteristics

			Maximum troponin I; Above median n = 74		Maximum creatine kinase-MB; Above median n = 74		Q-wave infarction n = 51		Left ventricular ejection fraction (LVEF) ≤ 50% n = 79	
	
	n	%	OR^1 ^ (95% CI^2^)	p	OR^1 ^ (95% CI^2^)	p	OR^1 ^ (95% CI^2^)	p	OR^1 ^ (95% CI^2^)	p
Age	58.1 (mean)	± 8.7 (SD^3^)	0.98 (0.94-1.01)	0.22	0.98 (0.94-1.01)	0.18	1.02 (0.98-1.06)	0.45	1.01 (0.97-1.05)	0.56
Female sex	39	26.5	0.69 (0.33-1.45)	0.33	0.80 (0.38-1.66)	0.54	1.08 (0.50-2.31)	0.85	2.42 (1.11-5.27)	0.03
Immigrant	17	11.6	2.63 (0.88-7.90)	0.08	2.63 (0.88-7.90)	0.08	1.80 (0.65-4.99)	0.26	0.56 (0.20-1.57)	0.27
Educational level less than 9 years	42	28.6	0.50 (0.24-1.04)	0.06	0.50 (0.24-1.04)	0.06	1.07 (0.50-2.25)	0.87	0.71 (0.35-1.45)	0.35
History of myocardial infarction	18	12.2	0.76 (0.28-2.06)	0.59	0.45 (0.16-1.27)	0.13	0.34 (0.09-1.23)	0.10	2.48 (0.84-7.36)	0.10
Previous revascularization^4^	17	11.6	0.37 (0.12-1.11)	0.08	0.18 (0.05-0.65)	0.01	0.37 (0.10-1.34)	0.13	1.67 (0.58-4.79)	0.34
Treatment for hypertension	65	44.2	1.15 (0.60-2.21)	0.67	0.92 (0.48-1.77)	0.81	1.52 (0.77-3.01)	0.23	1.26 (0.65-2.42)	0.49
Treatment for hyperlipidemia	31	21.1	0.91 (0.41-2.00)	0.81	0.55 (0.25-1.24)	0.15	1.05 (0.46-2.40)	0.92	1.48 (0.66-3.32)	0.34
Treatment for angina pectoris	29	19.7	0.44 (0.19-1.04)	0.06	0.37 (0.15-0.87)	0.02	0.99 (0.42-2.32)	0.98	2.72 (1.11-6.62)	0.03
Treatment for diabetes mellitus	22	15.0	0.51 (0.20-1.30)	0.16	0.79 (0.32-1.97)	0.62	0.86 (0.33-2.27)	0.76	1.62 (0.63-4.12)	0.32
BMI	27.5 (mean)	± 4.5 (SD^3^)	1.01 (0.94-1.09)	0.76	1.02 (0.94-1.09)	0.68	0.98 (0.91-1.06)	0.65	1.03 (0.96-1.11)	0.38
Living alone	36	24.5	0.63 (0.29-1.35)	0.23	1.14 (0.54-2.42)	0.74	1.49 (0.69-3.22)	0.31	0.82 (0.39-1.74)	0.61
Currently smoking	65	44.2	1.44 (0.75-2.76)	0.28	2.26 (1.16-4.39)	0.02	1.72 (0.87-3.41)	0.12	1.13 (0.59-2.16)	0.72
Presently not employed or professionally active	74	50.3	0.70 (0.37-1.34)	0.28	0.78 (0.41-1.50)	0.46	1.49 (0.75-2.96)	0.25	1.78 (0.92-3.43)	0.09
Moderate to severe level of depression (BDI ≥ 17)	15	10.2	1.14 (0.39-3.33)	0.81	1.14 (0.39-3.33)	0.81	0.66 (0.20-2.18)	0.49	6.50 (1.41-29.94)	0.02

Distribution and associations with four different indicators of severity of myocardial infarction (n = 147).

^1^Odds Ratio; ^2^Confidence Interval; ^3^Standard Deviation; ^4^PCI (Percutaneous Coronary Intervention) or CABG (Coronary Artery Bypass Graft)

### Univariate analyses

Background factors and clinical characteristics were analyzed in association with each indicator of severity. No factor showed statistically significant univariate associations with maximum troponin I levels above median or with a pathological Q-wave infarction. Current smoking was associated with CKMB-levels above median whereas previous revascularization and treatment for angina pectoris showed statistically significant associations with CKMB-levels below median. Female sex, treatment for angina pectoris, and BDI-scores ≥ 17 were associated with a decreased LVEF.

We continued the analyses by investigating whether behavior in a stressful situation as measured by the serial CWT and personality dimensions were related to severity of disease (Table 2). The behavior in the test and the personality dimensions were each analyzed separately in relation to the four indices of severity. Behavior in the CWT was in these univariate analyses not associated with any severity indicator in a statistically significant manner. The difference in maximum troponin I-levels was statistically significant comparing patients in the extraversion dimension of personality. Patients scoring low on extraversion had higher maximum troponin I-levels than patients scoring high.

**Table 2:** Behavior in the serial CWT and personality-factors in relation to four indicators of severity of myocardial infarction (n = 147)

		Maximum troponin I		Maximum creatine kinase-MB		Q-wave infarction n = 51		Left ventricular ejection fraction (LVEF) ≤ 50% n = 79	
	
	n	mean	p-value^1^	mean	p-value^1^	%^2^	p-value^3^	%^2^	p-value^3^
Behavior in the CWT									
Adaptive	74	18.57		80.22		35.1		51.4	
Maladaptive	73	20.78	0.71	85.13	0.75	34.2	0.91	56.2	0.56
Neuroticism									
Low level	100	17.16		81.82		36.0		55.0	
High level	47	25.01	0.22	84.45	0.87	31.9	0.63	51.1	0.66
Extraversion									
Low level	45	29.58		102.58		44.4		57.8	
High level	102	15.29	0.03	73.87	0.08	30.4	0.10	52.0	0.51
Openness									
Low level	47	23.45		78.96		25.5		51.1	
High level	100	17.89	0.39	84.40	0.74	39.0	0.11	55.0	0.66
Agreeableness									
Low level	48	18.29		89.24		35.4		52.1	
High level	99	20.33	0.75	79.47	0.55	34.3	0.90	54.5	0.78
Conscientiousness									
Low level	52	23.57		98.55		34.6		48.1	
High level	95	17.53	0.33	73.97	0.13	34.7	0.99	56.8	0.31

^1 ^One-way ANOVA; ^2 ^Percentage within each category of behavior in the CWT or within each level of personality dimension; ^3 ^Pearson Chi-square.

### Multivariate analyses

Extraversion was chosen as the personality dimension to be analyzed in conjunction with adaptive behavior for the investigation of a possible association with severity of MI. Because we had four indicators of severity there were carried out four separate analyses. A maladaptive behavior in the serial CWT together with a low level in the personality dimension extraversion was associated with maximum levels above median of cardiac troponin I (OR 2.97, CI 1.08-8.20, p = 0.04) (Table 3). A statistically significant association between maximum CKMB-levels above median and a maladaptive behavior together with low scores on extraversion could also be established (OR 3.33, CI 1.12-9.93, p = 0.03). Current smoking (OR 2.36, CI 1.14-4.88, p = 0.02) and previous revascularization (OR 0.19, CI 0.04-1.00, p = < 0.05) did also show associations with CKMB-levels above median. No association was found between Q-wave infarctions and the combination maladaptive behavior and low scores on extraversion nor were any of the included background factors or clinical characteristics related to Q-wave infarctions. Female sex was associated with a decreased LVEF but no other associations could be discerned in the multivariate analyses.

**Table 3:** Results from multivariate analyses: Background, clinical, and psychosocial factors and their associations with four different indicators of severity of myocardial infarction, (n = 147).

Indicator of severity	Factor	OR^1^	95% C I^2^	p
				
Maximum troponin I-level; above median	Behavior in the CWT and level of extraversion			
	Adaptive behavior and high level of extraversion		Reference	
	Adaptive behavior and low level of extraversion	1.88	0.60-5.90	0.28^3^
	Maladaptive behavior and high level of extraversion	1.23	0.53-2.83	0.63^3^
	Maladaptive behavior and low level of extraversion	2.97	1.08-8.20	0.04^3^
	Formal education of < 9 years	0.48	0.21-1.08	0.08
	Immigrant	2.18	0.70-6.79	0.18
	Treatment for angina pectoris	0.52	0.17-1.62	0.26
	Previous revascularization^4^	0.63	0.15-2.72	0.54
				
Maximum creatine kinase-MB; above median	Behavior in the CWT and level of extraversion			
	Adaptive behavior and high level of extraversion		Reference	
	Adaptive behavior and low level of extraversion	1.26	0.40-4.01	0.70^3^
	Maladaptive behavior and high level of extraversion	1.10	0.46-2.61	0.83^3^
	Maladaptive behavior and low level of extraversion	3.33	1.12-9.93	0.03^3^
	Current smoking	2.36	1.14-4.88	0.02
	Previous revascularization^4^	0.19	0.04- < 1.00	0.05
	Formal education of < 9 years	0.50	0.21-1.15	0.10
	Immigrant	2.27	0.69-7.51	0.18
	Treatment for angina pectoris	0.71	0.22-2.33	0.58
				
Q-wave infarction	Behavior in the CWT and level of extraversion			
	Adaptive behavior and high level of extraversion		Reference	
	Adaptive behavior and low level of extraversion	1.31	0.43-4.02	0.64^3^
	Maladaptive behavior and high level of extraversion	0.73	0.31-1.75	0.48^3^
	Maladaptive behavior and low level of extraversion	1.65	0.65-4.18	0.30^3^
	History of MI	0.37	0.10-1.34	0.37
				
Left ventricular ejection fraction (LVEF) ≤ 50%	Behavior in the CWT and level of extraversion			
	Adaptive behavior and high level of extraversion		Reference	
	Adaptive behavior and low level of extraversion	1.15	0.35-3.80	0.82^3^
	Maladaptive behavior and high level of extraversion	1.14	0.49-2.64	0.76^3^
	Maladaptive behavior and low level of extraversion	1.06	0.39-2.91	0.91^3^
	Female sex	2.28	< 1.00-5.21	< 0.05
	Moderate to severe level of depression (BDI ≥ 19)	4.56	0.89-23.26	0.07
	Presently not employed or professionally active	1.62	0.81-3.27	0.17
	History of MI	2.05	0.55-7.59	0.28
	Treatment for angina pectoris	1.45	0.48-4.39	0.51
				

^1^Odds Ratio

^2^Confidence Interval

^3^In comparison with the reference stratum

^4^PCI (Percutaneous Coronary Intervention) or CABG (Coronary Artery Bypass Graft)

## Discussion

The results from this study suggest that patients with a maladaptive behavior in the serial CWT together with low scores on extraversion were the patients where the highest maximum levels of the two cardiac biomarkers troponin I and CKMB were registered in connection with the acute event. These associations were independent of sociodemographic factors, biomedical factors, and even moderate to severe levels of depression. To conclude that an association between severity and psychological factors was found is, however, premature because the relationship was not found when severity was defined as a Q-wave infarction or a decreased LVEF.

Psychosocial stress is both among laymen and professionals generally considered to contribute to adverse effects on the heart. Estimations from the INTERHEART-study including results from 52 different countries showed that psychosocial stressors collectively accounted for approximately 33% of the population attributable risk of AMI [4]. Our focus is on behavior in the stressful situation. Stress is commonly reported as a risk factor in association with CHD but it is a vague concept and is measured in many various manners [35]. When a stressor is identified, activations in the hypothalamic-pituitary-adrenocortical and sympathetic-adrenomedullary systems are heightened and followed by responses of the cardiovascular system [36]. The CWT does not measure cardiovascular reactivity per se but tells us how individuals actually behave when facing challenging tasks. A pathophysiological mechanism that may explain why individuals with a maladaptive behavior are exposed to an increased risk of CVD is that these individuals may be more likely to show elevated blood pressure levels when responding to stress.

The assumption that a maladaptive behavior in the face of challenging situations would be associated with a more severe infarction could, however, not be substantiated when maladaptive behavior was considered by itself. The relationship between maximum troponin I- and CKMB-levels above medians and maladaptive behavior was only found when the maladaptive behavior was present in conjunction with low levels of extraversion. This aspect of personality gives a measure of an individual's level of social interaction. Individuals scoring low could be characterized as reserved or introverted. A suggested pathway between disease and this specific personality-type is that individuals with low levels of extraversion have not been able to cultivate their social network and as a consequence lack the social support that could increase resilience to disease [37].

The finding that maladaptive stress-behavior together with low levels of extraversion was associated with higher levels of cardiac biomarkers at the time of the event should be compared with results from Denollet and colleagues [17,38,39] because there are some similarities regarding social interaction. They studied personality as a risk-factor in relation to CHD and found the Type D personality especially vulnerable to chronic distress and thereby to adverse health outcomes. The distinguished features of that personality-type are negative affectivity and social inhibition. The suggested pathogenic effects of social inhibition on disease is that social interaction is experienced as threatening to the socially inhibited individual who in such situations responds with increased sympathetic activity, elevated levels of catecholamines, increased heart rate, and increased blood pressure levels [39].

No association between maladaptive behavior in combination with personality and a decreased LVEF was found. In this aspect our results are not compatible with those from Denollet and Brutsaert [38]. They found that severity, defined as a decreased LVEF, in combination with Type D personality and younger age was associated with an increased risk of a cardiac event during a follow-up time of almost eight years. It should, however, be mentioned that momentary drops of > 5% in LVEF in response to laboratory stressors have been observed and studied in CAD-patients. Such drops can be prognostically significant because patients exhibiting such drops in response to stress were the ones most likely to experience a cardiac event during a four year follow-up [35].

Another discrepancy that appears when comparing results from our study with results from other studies concerns depression. Moderate to severe levels of depression as measured with the BDI was used as a covariate in our analyses of a possible association between stress-behavior, personality, and severity of MI. A univariate association between moderate to severe levels of depression and a decreased LVEF was observed but the association could not be confirmed in the multivariate analysis. This is noteworthy because depression has increasingly been shown to confer increased risk of negative prognosis following an MI. Psychological factors in association with severity of MI have rarely been studied but in a recent publication with a decreased LVEF as a measure of severity was found that the somatic/affective aspects as opposed to the more cognitive/affective aspects of depression were associated with severity and prognosis [40]. This could possibly serve as an explanation as to why we did not find an association between behavior in the CWT, personality and a decreased LVEF. Behavior in the serial CWT relies on perception which is an act of cognition [33].

Depression has strongly been related to MI and subsequent survival [41] and depression preceding the infarction has been related to worse cardiac failure [42]. Frasure-Smith and co-writers [43] found a prevalence of depression at about 32% in a population of 887 MI-patients that were interviewed concerning depression and social support seven days following the event. The proportion with moderate to severe signs of depression (as measured with the BDI) in our study cohort was 10.2% and consequently considerably smaller. A possible explanation is that our examination occurred with closer proximity to the event, a time when feelings of gratefulness for having been taken care of could be the most prominent emotion.

Neuroticism was perhaps the personality dimension that was expected to be associated with disease severity because its associations with anxiety, emotional instability, and depressiveness [24]. Anxiety as a separate emotion has been associated with an increased risk of incident MI in older men [44] and is estimated to be as high as 70-80% among patients that have experienced an acute cardiac event [45]. Our results did not indicate associations between severity and neuroticism which could be explained by the fact that the study cohort was substantially circumscribed because a majority of the SECAMI-patients were not fit to participate in the serial CWT.

The strength of the present study is the semi-experimental manner in which behavior in a stressful situation was measured which means that the way the situation is managed is not influenced by a retrospective memory bias. Shortcomings should also be mentioned. The first concerns reliability of results which cannot be considered conclusive because maladaptive behavior in conjunction with the personality dimension extraversion was not associated with all four measures of severity. The reasons for the lacking associations between behavior in the CWT and the personality extraversion on the one hand and a decreased LVEF or Q-wave infarction on the other can, of course, be ascribed to nonexistent relationships but can also be referred to the way behavior was categorized. Previous studies have been based on the norms obtained from the study "Men born in 1914" which is the only study where the serial CWT has been applied in an epidemiologic context and been validated as a prognostic risk factor in association with CVD. Categorization of behavior was in the present study necessarily based on behavior observed in the 180 patients that completed the serial CWT during their hospital stay which means that the norms can not be generalized to all populations. It is possible that categorization should have been based specifically on women by themselves and men by themselves or on specific age groups. That adjustment would, however, have left us with too small groups for multivariate analyses to become meaningful.

The study cohort was small because not all patients did consent to participate in the CWT and some felt too frail to finish the complete test. Certain selection effects were, thus, at play and could have contributed to the lack of an association between behavior and Q-wave infarctions or a decreased LVEF. However, the 253 patients that were excluded were compared with the included 147 and there could not be discerned any statistically significant differences regarding age or prevalence of maximum troponin I above median, maximum creatine kinase-MB above median, left ventricular ejection fraction (LVEF) ≤ 50% or q-wave infarctions (not in table). It should also be remembered that patients with an unstabilized condition and those above the age of 70 years had been excluded. Another criticism that should be pointed out was that amount of physical exercise was not asked about. Furthermore can be mentioned that approximately 30% of the population with incident MI die before reaching hospital [46].

There is also a need to point at some general methodological issues that entitles very careful interpretation of our results. The present study is by character explorative. We chose to investigate the relationship between maladaptive behavior in combination with five specific personality dimensions in association with four different indicators of severity of MI. These analyses showed that the combination of maladaptive behavior and low levels of extraversion could be associated with severity of disease. By making many comparisons without proper adjustment there is always a risk that the significant finding could be the result of chance alone. The combination of behavior in the serial CWT and level on extraversion was entered into the multivariate analyses together with those traditional cardiovascular risk-factors that had shown an association with the particular severity indicator with a p-level < 0.20 and were not chosen according to an a priori selected hypothesis. It is possible that this procedure allows too many confounders.

The severity indicators reflect different aspects of pathophysiology and four measures were chosen with the conception of gaining greater credibility if stress behavior and personality factors would have been associated with all four indicators. All indicators are relevant but because they are used as single entities and not combined in a joint measure as e. g. the PREDICT-score [47] they may not reflect true severity. Although biomarkers are specific, sensitive and their circulatory levels agree well with level of necrosis it is the subsequent survival following the acute event that is essential. The follow-up of the SECAMI-cohort aims at providing more definite answers on how psychosocial factors act on subsequent survival following an AMI.

## Conclusions

In this cohort of AMI-patients we wanted to investigate whether a maladaptive behavior in a stressful situation in combination with any of the personality dimensions Neuroticism, Extraversion, Openness, Conscientiousness, or Agreeableness would be associated with the severity of an MI. We found that a maladaptive behavior in the serial CWT together with low scores in the Extraversion-dimension was related to severity of disease when severity was defined as maximum levels above median of the cardiac biomarkers troponin I and CKMB. The association was not found when severity was defined as a Q-wave infarction or a decreased LVEF which makes it premature to state that there exist associations between behavior in stressful situations, personality dimensions, and severity of infarction. An implication of the results, however inconclusive, is that individuals with difficulties in adapting to stressful events could be exposed to an increased risk of a more severe disease but above all, these individuals should be identified at an even earlier stage in their disease.

## Competing interests

The authors declare that they have no competing interests.

## Authors' contributions

LAP participated in the design of the study, collected the material, performed the statistical analyses, and drafted the manuscript. MS participated in the design of the study and collected the material. GE, PT, and BH designed the study and revised the manuscript. All authors have read and approved the manuscript.

## Authors' information

LA-P: Department of Clinical Sciences, Lund University, Skåne University Hospital, Malmö, Sweden, and Department of Neurology, Skåne University Hospital, Malmö, Sweden.

MS: Department of Cardiology, Lund University, Skåne University Hospital, Malmö, Sweden.

GE: Department of Clinical Sciences, Lund University, Skåne University Hospital, Malmö, Sweden.

PT: Department of Cardiology, Lund University, Skåne University Hospital, Malmö, Sweden.

BH: Department of Clinical Sciences, Lund University, Skåne University Hospital, Malmö, Sweden.

## Pre-publication history

The pre-publication history for this paper can be accessed here:

http://www.biomedcentral.com/1471-2261/11/45/prepub
